# A Boot Brace for Elephantiasis Neuromatosa Associated with Neurofibromatosis Type 1

**DOI:** 10.1097/GOX.0000000000002353

**Published:** 2019-08-05

**Authors:** Akiko Yoshinaga, Susumu Saito

**Affiliations:** From the Department of Plastic and Reconstructive Surgery, Graduate School of Medicine and Faculty of Medicine, Kyoto University, Kyoto, Japan

## Sir,

Neurofibromatosis type 1 (NF-1) is a rare autosomal dominant disease that affects approximately 1 in 3,500 people.^1^ Clinical symptoms of NF-1 include a variety of cutaneous and skeletal abnormalities such as freckling and café-au-lait macules, multiple neurofibromas, and scoliosis. Plexiform neurofibroma is an uncommon variant of NF-1 characterized by deformed masses growing along the length of nerves and affecting surrounding tissues such as skin, muscle, and bone.^1^ Although rare, abnormal soft-tissue hypertrophy and bone dysplasia can occur in patients with plexiform neurofibroma. This condition is termed elephantiasis neuromatosa (EN).^2–4^ Changes in the vascular and lymphatic system, such as enlarged veins,^2^ dilated lymphatics,^3^ enlarged lymph nodes,^3,4^ and dermal backflow,^4^ have been observed in limbs enlarged by EN. Although surgery is the standard treatment for plexiform neurofibroma,^5^ conservative treatment might be an option, especially for more complicated cases such as EN. Here, we describe a custom boot-type brace that we used to treat EN.

**Fig. 1.**
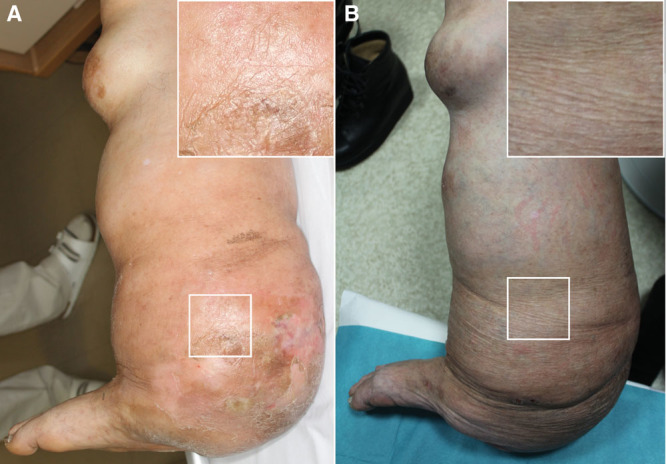
Changes in skin condition of the leg affected by elephantiasis neuromatosa with brace therapy. Photographs showing the condition of the skin on the leg at the time of infection (A) and 3 years after brace use began (B). A magnified image of the rectangular region in each photograph is shown in the corner inset.

A 59-year-old man was referred for evaluation of a giant mass in the right lower extremity from just below the knee to the foot that was diffusely swollen and drooping. He had previously been diagnosed with NF-1 based on clinical symptoms. The circumference of the right lower leg was up to 61.5 cm, compared to 33 cm on the left. The patient was a welder and the mass was frequently injured while walking around at his workplace. Surgery was not indicated because of the risk of injuries to the neurovascular bundles. Because the patient had a history of severe cellulitis and abscess formation (Fig. 1), we decided to apply a custom boot-type brace to protect the leg from injuries and avoid further infection.

**Fig. 2.**
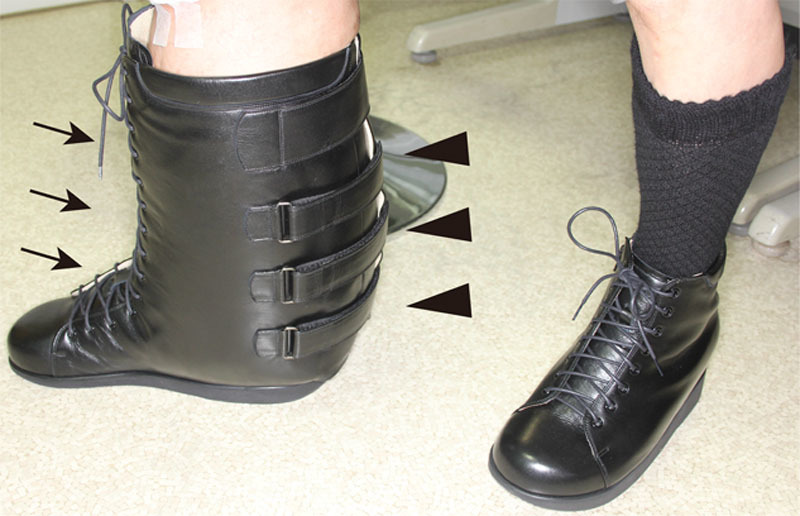
Custom boot-type brace. The brace is characterized by a double door–opening system, which is composed of an opening with shoelaces (arrows) in the front and an opening with hooks and loop fasteners (arrowheads) in the back.

Regarding brace design, we emphasize the following 2 points. One is to ensure a comfortable fit to the complicated form of the leg and the other is to ensure ease of putting on and taking off the brace because comfort and ease of use improve compliance. Because the leg was so heavy and large, a double door–opening system was used, which allowed the boot to be removed through the front or the back (Fig. 2). The fit was adjustable with shoelaces on the front, which facilitates controlling lower extremity edema. The opening at the back had hooks and loop fasteners for easy wearing. During 3 years of brace use, the patient did not have any recurrences of skin infection. Moreover, the skin became softer and smoother, although the size of the leg did not show any substantial decrease (Fig. 1).

An association between lymphostasis and lymphedema with extensive hyperproliferation of soft tissues in EN has been suggested.^3,4^ In the present case, both protection from injuries and continuous compression of the leg might have contributed to improvements in skin condition. We believe the double door–opening system could be optimal for these purposes. We hope that this report could become a guide for clinicians treating patients with EN.

## ACKNOWLEDGMENTS

The brace was manufactured by Kawabata Seisakusho. The materials used in the brace are as follows:outer surface, cow leather; lining, cow and pig leather; counter (shoe-sole base), vegetable tanned leather; toe box, cotton and styrene latex resin; heel, rubber and foam rubber; insole core, ethylene vinyl acetate (EVA); and insole cover, lightweight EVA, natural zeolite, and polyester fiber. We thank the patient for granting permission to publish this information.
